# Broad Range Screening of Vector-Borne Pathogens in Arctic Foxes (*Vulpes lagopus*) in Iceland

**DOI:** 10.3390/ani10112031

**Published:** 2020-11-04

**Authors:** Sándor Hornok, Kristin Mühldorfer, Nóra Takács, Regina Hofmann-Lehmann, Marina L. Meli, Miklós Gyuranecz, Ester R. Unnsteinsdóttir, Alex D. Greenwood, Gábor Á. Czirják

**Affiliations:** 1 Department of Parasitology and Zoology, University of Veterinary Medicine, 1078 Budapest, Hungary; takacs.nora@univet.hu; 2 Department of Wildlife Diseases, Leibniz Institute for Zoo and Wildlife Research, 10315 Berlin, Germany; muehldorfer@izw-berlin.de (K.M.); greenwood@izw-berlin.de (A.D.G.); 3 Clinical Laboratory, Department of Clinical Diagnostics and Services, and Center for Clinical Studies, Vetsuisse Faculty, University of Zurich, 8057 Zurich, Switzerland; rhofmann@vetclinics.uzh.ch (R.H.-L.); mmeli@vetclinics.uzh.ch (M.L.M.); 4 Institute for Veterinary Medical Research, Centre for Agricultural Research, 1143 Budapest, Hungary; m.gyuranecz@gmail.com; 5 Ecology and Consultancy Department, Icelandic Institute of Natural History, 210 Garðabær, Iceland; Ester.R.Unnsteinsdottir@ni.is; 6 Department of Veterinary Medicine, Freie Universität Berlin, 14163 Berlin, Germany

**Keywords:** arctic fox, Iceland, vector-borne bacteria, vector-borne protozoan parasites, climate change

## Abstract

**Simple Summary:**

The arctic fox is the only native terrestrial mammal in Iceland. The population comprises both “coastal” and “inland” fox ecotypes, with regard to food resources. Because of the relatively low biodiversity within arctic ecosystems and the involvement of the species in both marine and terrestrial ecosystems, the Icelandic arctic fox population could serve as sentinels for the overall ecosystem health of Iceland. After screening the samples from 60 foxes for tick-/vector-borne pathogens, this study reports the near-absence (very low prevalence) of these pathogens in Icelandic arctic foxes in 2011–2012. Taking into account the broad range of target microorganisms analyzed here, as well as the warming climate and increasing presence of the vector *Ixodes ricinus* in Iceland, our results will be very useful as baseline data for comparison in future monitoring of the emergence of ticks and tick-borne diseases in this country.

**Abstract:**

The arctic fox (*Vulpes lagopus*) is the only native terrestrial mammal in Iceland. While red foxes (*V. vulpes*) are known to be epidemiologically important carriers of several vector-borne pathogens in Europe, arctic foxes have never been evaluated in a similar context on this continent. This has become especially relevant in the last decade, considering the establishing populations of the tick species *Ixodes ricinus* in Iceland. In this study, liver DNA extracts of 60 arctic foxes, hunted between 2011–2012, were molecularly screened for vector-borne protozoan parasites (*Trypanosomatidae*, *Babesia*, *Theileria*, *Hepatozoon*) and bacteria (*Anaplasma*, *Ehrlichia*, *Rickettsia*, *Borrelia*, hemotropic *Mycoplasma*). One sample was real-time qPCR positive for *Anaplasma phagocytophilum*, though this positivity could not be confirmed with sequencing. Samples were negative for all other tested vector-borne pathogens. Results of this study indicate that, except for *A. phagocytophilum*, Icelandic arctic foxes were apparently “not yet infected” with vector-borne pathogens in 2011–2012, or their infections were “below the detection limit” of applied methods. Taking into account the broad range of target microorganisms analyzed here, as well as the warming climate and increasing presence of the vector *I. ricinus* in Iceland, our results will be very useful as baseline data for comparison in future monitoring of the emergence of ticks and tick-borne diseases in this country.

## 1. Introduction

The arctic fox (*Vulpes lagopus*) is a small size (ca. 3–5 kg) generalist predator species with circumpolar distribution, associated primarily with arctic and tundra habitats. In Iceland, the arctic fox is the only native terrestrial mammal, divided into coastal and inland ecotypes [1]. While coastal ecotype foxes mainly feed on sea birds and eggs, invertebrates, and marine mammal carcasses, the inland foxes feed on ptarmigans, migrating waterfowl, eggs, reindeer and livestock carcasses, and wood mice [2].

The closest relative of the arctic fox in the Palearctic region is the red fox (*V. vulpes*), and in northern countries, these two species show partly overlapping geographical range and niche [3]. The red fox is widespread in Europe and is known to harbor a broad range of pathogens transmitted by hard ticks (Acari: *Ixodidae*) and other blood-sucking arthropod vectors [4,5,6]. This implies that red foxes participate in the natural transmission cycle of vector-borne pathogens but may also be used as sentinels for epidemiological surveillance of associated disease risks. On the other hand, a similar epidemiological role has not been postulated for the arctic fox, most likely because they were not found to be tick-infested in early studies [7]. However, this scenario may change with the warming climate, as increasing tick abundance has recently been experienced in Iceland [8].

Eight hard tick species have been reported in Iceland [9], among which *Ixodes ricinus* has the highest veterinary-medical importance in Europe [10]. Since the first record of *I. ricinus* in Iceland, dating back to 1967, its growing significance has been reported [8,9]. Apart from the import of this tick species by migratory birds, its host-questing activity on the vegetation is a likely indicator of established populations [8]. Importantly, arctic foxes are among the possible wildlife reproduction hosts of *I. ricinus* (i.e., feeding adults), while rodents, such as wood mice (*Apodemus sylvaticus*), can support larvae and nymphs.

Between 2012–2013, vector-borne pathogens were examined in 28 arctic foxes in Canada, and *Bartonella henselae*, *Mycoplasma haemocanis*, and *Ehrlichia canis* were detected [11]. However, despite the emergence of *I. ricinus* in Iceland, arctic foxes have not been screened for vector-borne pathogens in this country. Thus, the aim of the present study was to screen liver samples of 60 arctic foxes from Iceland for DNA of vector-borne protozoa (Euglenozoa: *Trypanosomatidae*; Apicomplexa: *Babesia*, *Theileria*, *Hepatozoon*) and bacteria (Proteobacteria: *Anaplasma*, *Ehrlichia*, *Rickettsia*, *Bartonella*; *Spirochaetes*: *Borrelia*; Tenericutes: hemotropic *Mycoplasma*), with emphasis on tick-borne pathogens.

## 2. Materials and Methods

All experimental procedures described in the materials and methods section were approved by the Internal Committee for Ethics and Animal Welfare of the Leibniz Institute for Zoo and Wildlife Research (permit #2010-01-03). All experiments were carried out in compliance with the approved guidelines of the Leibniz Institute for Zoo and Wildlife Research and the laws of Germany, Hungary, and Iceland.

Liver samples of 60 arctic foxes hunted between 2011 and 2012 in various inland and coastal regions of Iceland were used in this study. Hunting was covered by the government and managed by The Environmental Agency, under the wildlife act (64/1994) and animal welfare act (55/2013). Scientific use of the harvest was organized by the Icelandic Institute of Natural History, courtesy of The Icelandic Ministry for the Environment and Natural Resources. The animals included 40 adults (23 males, 17 females) and 20 juveniles (11 males, 9 females). Age determination of the arctic foxes was performed by X-ray measurements based on dental pulp cavity volume and cementum analysis. Juveniles were defined as younger than 1 year and adults as older than 1 year of age [12]. Carcasses were stored at −20 °C until necropsy was performed.

During necropsy, approximately 10 g of liver tissue was sampled and then stored at −80 °C until further analysis. The liver was chosen as the target organ, because it contains blood from both the portal and systemic circulation and is rich in cells of the reticuloendothelial system, i.e., components that are the most likely to contain the DNA of hemotropic, vector-borne microorganisms. Ectoparasites were not seen on the foxes post-mortem at the time of sample collection.

DNA was extracted from liver samples, using the NucleoSpin Tissue kit (Macherey-Nagel GmbH, Düren, Germany), individually from each fox. A total of 100 µL of homogenized tissue was digested with Proteinase-K overnight at 56 °C and prepared as described in the user manual. Quality and quantity of extracted DNA were assessed by ND-1000 UV-Vis Spectrophotometer (Nanodrop Technologies, Wilmington, DE, USA) measurements.

All 60 DNA extracts were screened for a broad range of tick-/vector-borne pathogens with PCRs summarized in the Supplementary Material (Table S1). For *Anaplasma phagocytophilum*, the assay consisted of 40 cycles and the results were regarded as positive if the threshold cycle (Ct) value was below 39. The detection limit of this qPCR was a 0.125 ratio (one-eighths) of an *A. phagocytophilum* infected cell [13]. For Sanger sequencing of *A. phagocytophilum* from the real-time qPCR-positive sample, two conventional PCRs were attempted, the first by using the msp2 primers and annealing temperature, as in method “A” (Supplementary Materials, Table S1), and the second to amplify part of the heat shock chaperonin GroEL gene [14]. The latter PCR was repeated, but no sequenceable product was obtained.

## 3. Results and Discussion

Out of 60 samples, one adult female arctic fox from the costal ecotype was positive for *A. phagocytophilum*-specific real-time qPCR (Ct = 36.71; sequencing was repeatedly not successful). In line with this finding, red foxes have been reported to carry *A. phagocytophilum* DNA in at least 10 European countries [4,5]. *Anaplasma phygocytophilum* (Rickettsiales: Anaplasmataceae) is the causative agent of granulocytic anaplasmosis in multiple hosts (including humans) and tick-borne fever in ruminants [15]. In Europe, *A. phagocytophilum* is transmitted by *I. ricinus*, the tick-species increasingly recognized to affect animals in Iceland [8]. Nevertheless, to the best of our knowledge, this is the first molecular evidence of *A. phagocytophilum* in arctic foxes and the first autochthonous occurrence of this tick-borne species in Iceland. However, since this real-time qPCR positivity for *A. phagocytophilum* could not be confirmed with sequencing, probably because of the low amount of DNA, as reflected by the high Ct value in the more sensitive real-time qPCR, further studies are needed to verify the presence of this species in arctic foxes in Iceland. Although seropositivity to *A. phagocytophilum* has already been reported in humans in Iceland, in the absence of published data on the autochthonous occurrence of its vector in the country, this finding was explained by exposure during travel abroad [16].

All 60 samples were negative or contained DNA below the PCR detection limit for other tested vector-borne pathogens. Taking into account that red foxes are known to harbor a broad range of the vector-borne protozoa and bacteria targeted here (e.g., in northern Europe [5]; in southern Europe [4,6]), PCR-negativity of most samples implies that the warming climate of Iceland has not entailed changes of similar magnitude in the distribution of tick-borne pathogens in 2011–2012, as reported in the same period from Scandinavia [17]. This is further confirmed by the absence of any ticks on the 60 foxes analyzed here, as well as on 315 arctic foxes examined in 2015–2016 [8].

Unlike what is shown here, arctic foxes were reported to harbor *Bartonella* spp., *M. haemocanis*, and *E. canis* in northern Canada [11,18]. However, in the latter case, the majority of positive samples were flea-borne bartonellae and not tick-borne pathogens. At least three rodent species are known to occur in Iceland [19], but their mite and flea species are usually not shared with foxes [20]. Despite this, during prey consumption, the role of temporary infestation of arctic foxes with these non-host specific ectoparasites, in the transmission of vector-borne pathogens, should not be discounted [18]. Nevertheless, results of the present study suggest that different spectra of vector-borne bacteria might emerge in arctic foxes in Iceland compared to Canada.

## 4. Conclusions

The above results provide baseline data on the near-absence (very low prevalence) of tick-/vector-borne pathogens in Icelandic arctic foxes in 2011–2012. Already observed and foreseeable climate changes in Iceland (milder winter, warmer spring and autumn [8]) will most likely promote multi-focal establishment of *I. ricinus* and possibly other tick species in this country, with increased risks of autochthonous transmission of tick-borne pathogens [8]. This warrants continuous monitoring of emerging vector-borne pathogens in Iceland, similar to all arctic ecosystems. Taking into account the broad range of target microorganisms analyzed here (for which red foxes are known to be susceptible), as well as the warming climate and increasing presence of *I. ricinus* in Iceland, our results will be very useful as baseline data for comparison in future monitoring of the emergence of ticks and tick-borne diseases in this country.

## Supplementary Materials

The following are available online at https://www.mdpi.com/2076-2615/10/11/2031/s1. Table S1: Details of the screening methods used in this study, listed according to target groups of vector-borne pathogens.
