# Supplementary material for: Broad Range Screening of Vector-Borne Pathogens in Arctic Foxes (Vulpes lagopus) in Iceland
Source: Animals (Basel). 2020 Nov 4;10(11):2031. doi: 10.3390/ani10112031 (PMC7694187; doi:10.3390/ani10112031)
Supplement: Supplementary file 1 [file animals-10-02031-s001.pdf]

Table 1. Screening methods used in this study, listed according to target groups of vector-borne pathogens (A–J: bacteria, K–M: protozoa).

PCR	Target Group	Target Gene	Primers, Probes (5'–3')	Initial Denaturation	Cycle Denaturation	Cycle Annealing	Cycle Extension	Final Extension	Cycles
A	**Anaplasmataceae**	16S rRNA	EHR16SD (GGT ACC YAC AGA AGA AGT CC)<br>EHR16SR (TAG CAC TCA TCG TTT ACA GC)	95 °C, 10 min	95 °C, 30 s	55 °C, 30 s	72 °C, 45 s	72 °C, 5 min	40
B	***Anaplasma phagocytophilum***	msp2	ApMSP2f (ATG GAA GGT AGT GTT GGT TAT GGT ATT)<br>ApMSP2r (TTG GTC TTG AAG CGC TCG TA)<br>ApMSP2p (FAM-TGG TGC CAG GGT TGA GCT TGA GAT TG–TAMRA)	95 °C, 20 s	95 °C, 3 s	60 °C, 30 s		-	40
C	***Ehrlichia canis***	16S rRNA	Ec.139f (ATG GCT ATT CCG TAC TAC TAG GTA GAT TC)<br>Ec.32r (CAT GCA AGT CGA ACG GAC AAT)<br>Ec.61p (6-FAM-TCT GCC ACT AAC AAT TTC CTA TAG CCA GAG GC–TAMRA)	95 °C, 10 min	95 °C, 10 min	95 °C, 10 min			45
D	**Rickettsiaceae**	gltA	CS-F (TCG CAA ATG TTC ACG GTA CTT T)<br>CS-R (TCG TGC ATT TCT TTC CAT TGT G)<br>CS-P (FAM-TGC AAT AGC AAG AAC CGT AGG CTG GAT G-BHQ–1)	95 °C, 10 min	95 °C, 10 min	95 °C, 10 min		-	45
E	**Rickettsiaceae**	gltA	RpCS877p (GGG GGC CTG CTC ACG GCG G)<br>RpCS1258n (ATT GCA AAA AGT ACA GTG AAC A)	95 °C, 5 min	95 °C, 20 s	48 °C, 30 s	72 °C, 1 min	72 °C, 5 min	40
F	***Borrelia burgdorferi* s.l.**	flagellin	B.398f (GGG AAG CAG ATT TGT TTG ACA)<br>B.484r (ATA GAG CAA CTT ACA GAC GAA ATT AAT AGA)<br>B.421p (FAM-ATG TGC ATT TGG TTA TAT TGA GCT TGA TCA GCA A–TAMRA)	95 °C, 10 min	95 °C, 15 s	60 °C, 1 min		-	40/45
G	**haemoplasmas**	16S rRNA	Sybr_For (AGC AAT RCC ATG TGA ACG ATG AA)<br>Sybr_Rev1 (TGG CAC ATA GTT TGC TGT CAC TT)<br>Sybr_Rev2 (GCT GGC ACA TAG TTA GCT GTC ACT)	95 °C, 10 min	95 °C, 15 s	60 °C, 1 min		-	40
H	***Mycoplasma haemocanis***	16S rRNA	MycCatNewF (GAA AGT CTG ATG GAG CAA TAC CAT)<br>MycCatNewR (CTG GCA CAT AGT TWG CTG TCA CTT A)<br>Mhf-MGB (VIC-AGT ACT ATC ATA ATT ATC CCT CG–MGB)	95 °C, 10 min	95 °C, 15 s	60 °C, 1 min		-	45
I	***Mycoplasma haematoparvum***	16S rRNA	MycDogF (GAA AGT CTG ATG GAG CAA TAC CAC)<br>MycCatNewR (CTG GCA CAT AGT TWG CTG TCA CTT A)<br>CMhm-MGB (6-FAM-AAG GCT TAA TCA TTT CCT–MGB)	95 °C, 10 min	95 °C, 15 s	60 °C, 1 min		-	45
J	***Bartonella* spp.**	16S-23S ITS	BA325s (CTT CAG ATG ATG ATC CCA AGC CTT CTG GCG)	95 °C, 5 min	94 °C, 30 s	65 °C, 30 s	72 °C, 50 s	72 °C, 5 min	40

			BA1100as (GAA CCG ACG ACC CCC TGC TTG CAA AGC A)						
K	**Trypanosomatidae**	18S rRNA (nested PCR)	External primers: TRY927F (GAA ACA AGA AAC ACG GGA G) TRY927R (CTA CTG GGC AGC TTG GA)	94 °C, 3 min	94 °C, 30 s	58 °C, 60 s	72 °C, 60 s	72 °C, 7 min	30
			Internal primers: SSU561F (TGG GAT AAC AAA GGA GCA) SSU561R (CTG AGA CTG TAA CCT CAA AGC)	94 °C, 3 min	94 °C, 30 s	56 °C, 30 s	72 °C, 60 s	72 °C, 7 min	30
L	***Hepatozoon* spp.**	18S rRNA	HepF (ATA CAT GAG CAA AAT CTC AAC) HepR (CTT ATT ATT CCA TGC TGC AG)	95 °C, 5 min	95 °C, 40 s	57 °C, 40 s	72 °C, 60 s	72 °C, 7 min	35
M	**Piroplasms**	18S rRNA (nested PCR)	External primers: BTF1 (GGC TCA TTA CAA CAG TTA TAG) BTR1 (CCC AAA GAC TTT GAT TTC TCT C)	94 °C, 3 min	94 °C, 30 s	58 °C, 60 s	72 °C, 60 s	72 °C, 7 min	30
			Internal primers: BTF2 (CCG TGC TAA TTG TAG GGC TAA TAC) BTR2 (GGA CTA CGA CGG TAT CTG ATC G)	94 °C, 3 min	94 °C, 30 s	62 °C, 40 s	72 °C, 60 s	72 °C, 7 min	30

References

A – Hornok, S., Földvári, G., Elek, V., Naranjo, V., Farkas, R., de la Fuente, J. Molecular identification of *Anaplasma marginale* and rickettsial endosymbionts in blood-sucking flies (Diptera: Tabanidae, Muscidae) and hard ticks (Acari: Ixodidae). *Vet Parasitol* **2008**, *154*, 354–359.

B – Hornok, S., Meli, M.L., Gönczi, E., Halász, E., Takács, N., Farkas, R., Hofmann-Lehmann, R. Occurrence of ticks and prevalence of *Anaplasma phagocytophilum* and *Borrelia burgdorferi* s.l. in three types of urban biotopes: forests, parks and cemeteries. *Ticks Tick Borne Dis* **2014**, *5*, 785–789.

C – Foley, J., Drazenovich, N., Leutenegger, C.M., Chomel, B.B. Association between polyarthritis and thrombocytopenia and increased prevalence of vectorborne pathogens in Californian dogs. *Vet Rec* **2007**, *160*, 159–162.

D – Boretti, F.S., Perreten, A., Meli, M.L., Cattori, V., Willi, B., Wengi, N., Hornok, S., Honegger, H., Hegglin, D., Woelfel, R., Reusch, C.E., Lutz, H., Hofmann-Lehmann, R. Molecular investigations of *Rickettsia helvetica* infection in dogs, foxes, humans and *Ixodes* spp. ticks. *Appl Environ Microbiol* **2009**, *75*, 3230–3237.

E – Roux, V., Rydkina, E., Eremeeva, M., Raoult, D. Citrate synthase gene comparison, a new tool for phylogenetic analysis, and its application for the rickettsiae. *Int J Syst Bacteriol* **1997**, *47*, 252–261.

F – Leutenegger, C.M., Pusterla, N., Mislin, C.N., Weber, R., Lutz, H. Molecular evidence of coinfection of ticks with *Borrelia burgdorferi* sensu lato and the human granulocytic ehrlichiosis agent in Switzerland. *J Clin Microbiol* **1999**, *37*, 3390–3391.

G – Willi, B., Meli, M.L., Lüthy, R., Honegger, H., Wengi, N., Hoelzle, L.E., Reusch, C.E., Lutz, H., Hofmann-Lehmann, R. Development and application of a universal Hemoplasma screening assay based on the SYBR green PCR principle. *J Clin Microbiol* **2009**, *47*, 4049–4054.

H, I – Wengi, N., Willi, B., Boretti, F.S., Cattori, V., Riond, B., Meli, M.L., Reusch, C.E., Lutz. H., Hofmann-Lehmann, R. Real-time PCR-based prevalence study, infection follow-up and molecular characterization of canine haemotropic mycoplasmas. *Vet Microbiol* **2008**, *126*, 132–141.

J – Maggi, R.G., Diniz, P.P., Cadena, M.B., Breitschwerdt, E.B. The International Canine Vector-Borne Disease Symposium; April 18th-20th, 2006. Billesley, Alcester, UK; 2006. The Use of Molecular diagnostic Techniques to detect *Anaplasma*, *Bartonella* and *Ehrlichia* Species in Arthropods or Patiens; pp. 9-14.

K – Noyes, H.A., Stevens, J.R., Teixeira, M., Phelan, J., Holz, P. A nested PCR for the ssrRNA gene detects *Trypanosoma binneyi* in the platypus and *Trypanosoma sp.* in wombats and kangoroos in Australia. *Int J Parasitol* **1999**, *29*, 331-339.

L – Inokuma, H., Okuda, M., Ohno, K., Shimoda, K., Onishi, T. Analysis of the 18S rRNA gene sequence of a Hepatozoon detected in two Japanese dogs. *Vet Parasitol* **2002**, *106*, 265–271.

M – Jefferies, R., Ryan, U.M., Irwin, P.J. PCR-RFLP for the detection and differentiation of the canine piroplasm species and its use with filter paper-based technologies. *Vet Parasitol* **2007,** *144*, 20-27.
